# NonImmune hemolytic anemia secondary to vitamin B12 deficiency—A case report

**DOI:** 10.1002/ccr3.9514

**Published:** 2024-10-25

**Authors:** Ashish Tamang, Prakash Sapkota, Ram Bahadur Gurung, Abiral Subedi, Chhavi Sachdeva

**Affiliations:** ^1^ Kathmandu University School of Medical Sciences Dhulikhel Hospital Dhulikhel Nepal; ^2^ Gastroenterology and Interventional Endoscopy Unit, Department of Internal Medicine Kathmandu University School of Medical Sciences, Dhulikhel Hospital Dhulikhel Nepal

**Keywords:** anemia, cobalamin, endoscopy, gastroenterology, hemolysis, vitamin B12 deficiency

## Abstract

This case report emphasizes the crucial need to include vitamin B12 deficiency in the differential diagnosis of hemolytic anemia, despite its rarity as a presentation. The case illustrates that non‐immune hemolytic anemia can occur secondary to severe vitamin B12 deficiency, which can be effectively treated with vitamin B12 supplementation. Early recognition and comprehensive evaluation are essential for identifying this uncommon yet significant cause of hemolysis, ensuring prompt and appropriate treatment to improve patient outcomes.

## 1 INTRODUCTION

Vitamin B12 deficiency is a common condition with diverse clinical manifestations, ranging from mild anemia to severe neurological deficits.^1^, ^2^, ^3^ While the deficiency is typically associated with macrocytic anemia, recent studies have highlighted hemolysis as a potential, albeit atypical, presentation.^4^, ^5^ This case report presents a unique instance of a patient diagnosed with non‐immune hemolytic anemia, where an extensive evaluation ruled out common causes of hemolysis, leading to the diagnosis of vitamin B12 deficiency. The patient's condition improved significantly following treatment with vitamin B12 supplements. This case underscores the importance of considering vitamin B12 deficiency in the differential diagnosis of hemolytic anemia and highlights the potential clinical implications of this uncommon presentation.^4^, ^5^, ^6^ The clinical implications of vitamin B12 deficiency are vast and can lead to physical, neurological, and psychological symptoms if it is not treated.^1^, ^2^

## 2 CASE HISTORY AND EXAMINATION

A 41‐year‐old male patient with a known history of hypertension for the past 4 years presented with abdominal pain in the left and right lower quadrants. The pain was stabbing in nature, non‐radiating, and not severe enough to interfere with his daily activities. The patient had been experiencing this pain for the past month. He also reported instances of hematochezia occurring three times per week for the past month. The patient described the blood as fresh and noticed it after defecation; there was no melena. The patient also reported intermittent fever, with the last episode occurring 3 days before the consultation which was relieved by paracetamol. Additionally, the patient reported weight loss, back pain, chest pain, shortness of breath, and paresthesia in his palms and soles. He also reported instances of penile bleeding post‐coitus. There was no history of constipation, diarrhea or abdominal distension.

The initial laboratory findings [Table 1] reflected severe anemia (hemoglobin: 6.0 gm/dl) and elevated levels of lactate dehydrogenase (LDH) (16,286 U/L), ferritin (519.98 ng/mL), and vitamin B12 deficiency (<83 pg/mL). Peripheral blood smear displayed normocytic normochromic cells with anisocytosis and macrocytes. Notably, there was indirect bilirubinemia, marked by an elevated LDH, indicative of hemolysis.

**TABLE 1:** Blood parameters before treatment.

Parameters	Value	Normal
Total leukocyte count (× 10^3/μL)	4.5	4.0–11.0
Differential leukocyte count (DLC)		
Neutrophils %	53	40–75
Lymphocytes %	44	20–45
Eosinophils %	01	1–6
Monocytes %	02	2–8
Basophils %	00	0–1
Hemoglobin (gm/dl)	6.0	13–17
Platelet count (× 10^3/μL)	164	150–450
(Packed cell volume) PCV	21	40–50
Sodium (mmol/L)	141	135–148
Potassium (mmol/L)	3.7	3.5–5.3
Urea (mg/dl)	15	10–45
Creatinine (mg/dl)	0.5	0.6–1.3
Random blood glucose (mg/dl)	85	60–150
Prothombin time (seconds)	12	11–13.5
International normalized ratio (seconds)	1.0	0.9–1.1
Aspartate aminotransferase (IU/L)	139.0	5.0–40.0
Alanine aminotransferase (IU/L)	60	5.0–40.0
Alkaline phosphatase(U/L)	46	<115
Total bilirubin (mg/dL)	2.5	0.3–1.4
Direct bilirubin (mg/dL)	0.6	<0.5
Reticulocytes %	0.4	0.5%–2.5%
Mean corpuscular volume(fl)	114	80–100
Occult blood	Negative	
Vitamin B12 (pg/mL)	<83	187–883
Lactate dehydrogenase (U/L)	16,286	207–414
Coombs test	Negative	
Ferritin (ng/mL)	519.98	21.81–274.66
Folate (ng/mL)	14.0	3.1–20.5
Total iron‐binding capacity (mcg/dL)	305.0	Male: 261–462 Female: 265–497
Serum iron (mcg/dL)	212	65–175
Peripheral blood smear	RBC	Normocytic Normochromic with anisocytosis. Macrocytes(+)
	WBC	Normal in number and morphology. No atypical cells seen.
	Platelets	Markedly increased in number

**FIGURE 1:**
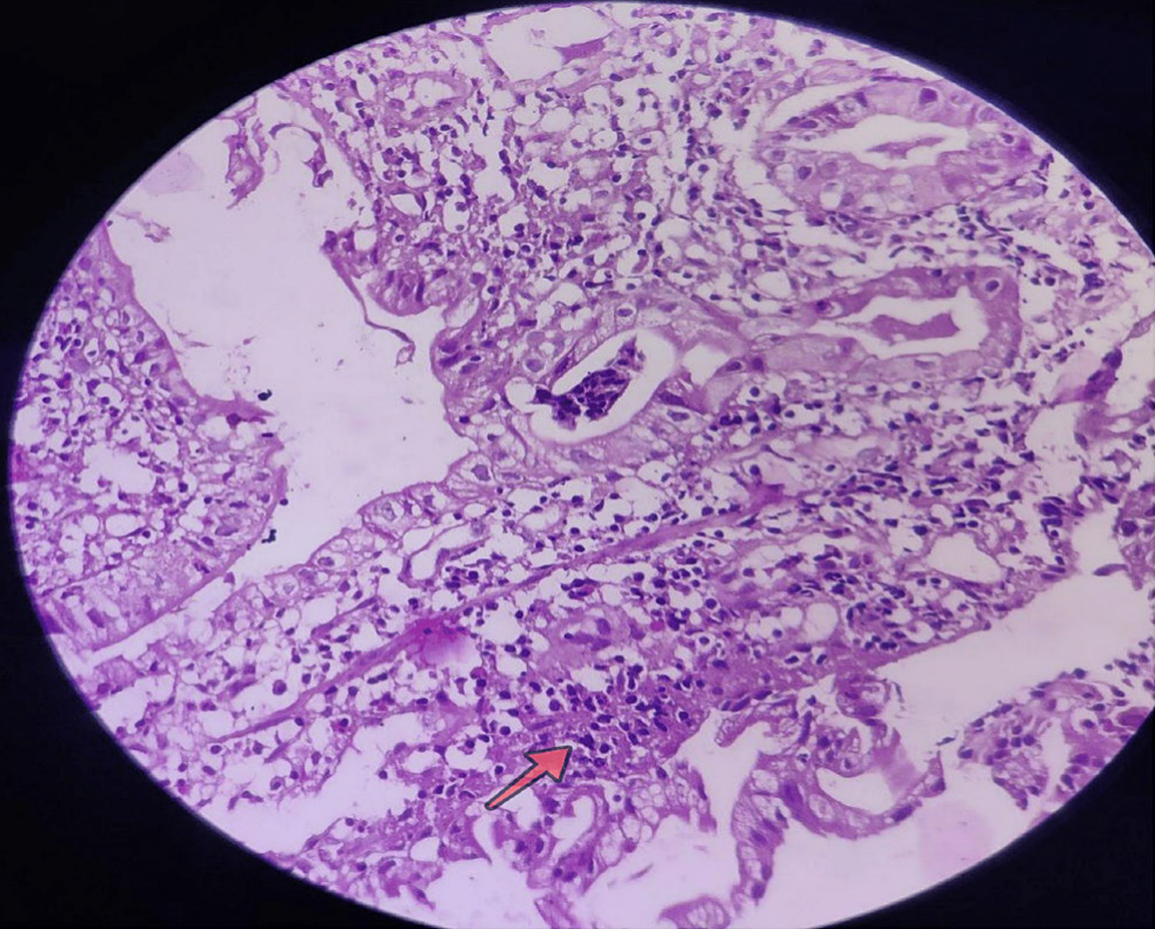
Histologic findings from terminal ileum showing focal cryptitis interspersed with goblet cells, chronic inflammatory cell infiltrates, aggregates of lymphocytes, few blood vessels and areas of hemorrhage neutrophilic infiltration (shown by arrow).

An upper gastrointestinal endoscopy revealed hyperemic mucosa in the antrum suggestive of antral gastritis. Biopsies were taken from the stomach, duodenum, terminal ileum, colon, and rectum ileum. The pathology report revealed ileal villus and glands lined by columnar epithelium with focal cryptitis interspersed with goblet cells with moderate chronic inflammatory cell infiltrates, aggregates of lymphocytes, few blood vessels and areas of hemorrhage in lamina propria [Figure 1]. This confirmed chronic, focal active ileitis in the terminal ileum.

The patient underwent a comprehensive treatment plan, receiving vitamin B12 1000 mcg intramuscularly weekly for 3 months alongside a proton pump inhibitor. Additionally, due to severe anemia, the patient received 2 pints of PRBC (Packed Red Blood Cells) to correct his hemoglobin levels.

Following 3 months of therapy, a remarkable improvement in the patient's condition was noted.

There was a marked improvement in hemoglobin levels from 6.0 gm/dL to 12.1 gm/dL with a significant rise in vitamin B12 [Table 2]. Additionally, the normalization of indirect bilirubinemia, indicated by the reduction in LDH levels signifies the effective treatment.

**TABLE 2:** Blood parameters after 3 months of treatment.

Parameters	Value	Normal
Total Leukocyte count (× 10^3/μL)	9.8	4.0–11.0
Differential Leucocytes Count		
Neutrophil%	73	40–75
Lymphocytes%	22	20–45
Monocytes%	04	2–8
Eosinophils%	01	1–6
Basophils%	00	0–1
Reticulocytes%	0.5	
Platelets Count (x 10^3)	405	150–450
Hemoglobin (g/dL)	12.1	13–17
Mean corpuscular volume (fl)	82	80–100
Ferritin (ng/mL)	80.11	Male: 18.2–341.2 Female (<45 years): 4.0–104.2 Female (>45 years): 4.9–232.3
Lactate Dehydrogenase (U/L)	354	207–414
Vitamin B12 (pg/mL)	>2000	187–883
Peripheral Blood Smear	RBC	Normocytic Normochromic
	WBC	Normal in number and morphology. Some neutrophils show toxic granules. No atypical cells seen.
	Platelets	Adequate in number

## 3 METHODS

The patient underwent a comprehensive assessment to identify the cause of hemolytic anemia. This involved considering various differential diagnoses, including inflammatory bowel disease (IBD) autoimmune conditions, infections, and nutritional deficiencies. Diagnostic investigations included a review of medical history, physical examination, complete blood count, peripheral smear examination, and serum levels of relevant markers such as vitamin B12, LDH, and ferritin. Gastrointestinal evaluations through endoscopy and colonoscopy were performed to rule out gastrointestinal pathology contributing to malabsorption. Biopsies were taken for histopathological examination. Treatment consisted of vitamin B12 supplementation and PRBC transfusion to correct severe anemia. The absence of classic IBD symptoms, infective markers, and the significant improvement with vitamin B12 therapy point towards a deficiency‐related inflammatory response rather than primary IBD, or other autoimmune conditions.

## 4 CONCLUSION AND RESULTS

This case report highlights the importance of considering vitamin B12 deficiency in patients presenting with unexplained anemia. Hemolytic anemia, though rare, is a potential manifestation of this deficiency. Early recognition and treatment with vitamin B12 supplementation significantly improved our patient's condition. Clinicians should be aware of this uncommon presentation to ensure prompt diagnosis and treatment. Further research is needed to understand the mechanisms of hemolysis in vitamin B12 deficiency.

## 5 DISCUSSION

The gastrointestinal tract plays a crucial role in the absorption of vitamin B12, an essential nutrient required for the production of red blood cells and DNA, as well as the proper functioning of the nervous system,^7^, ^8^ This absorption process relies on the presence of intrinsic factor, a protein produced in the stomach, which binds to vitamin B12 and facilitates its absorption in the terminal ileum.^9^ Any pathological changes in the terminal ileum, such as inflammation or ileitis, can disrupt this absorption process, leading to vitamin B12 deficiency and subsequent hematological abnormalities.^7^, ^8^ Similarly, autoimmune chronic atrophic gastritis, a condition characterized by the destruction of parietal cells and a reduction in intrinsic factors essential for vitamin B12 absorption, can also lead to vitamin B12 deficiency.^7^, ^8^

In our patient, the pathology report confirmed chronic, focal active ileitis in the terminal ileum [Figure 1]. This could have contributed to the malabsorption of vitamin B12, leading to the observed deficiency. These findings collectively suggest an ongoing inflammatory process in the terminal ileum of our patient, which could have disrupted the normal absorption of vitamin B12, leading to deficiency and subsequent hematological manifestations. The inflammation in the terminal ileum may have contributed to the malabsorption of vitamin B12, creating a vicious cycle of deficiency and further inflammation. Also, given the patient's symptoms, the histopathology findings and the patient's response to vitamin B12 supplementation suggest an alternative etiology for terminal ileitis.,^7^, ^8^

While the clinical and laboratory findings shared similarities with thrombotic microangiopathy (TMA), a detailed evaluation excluded TMA diagnosis. Crucially, the absence of schistocytes on peripheral smear, a key feature of TMA, and the patient's robust response to vitamin B12 supplementation were pivotal in differentiating the conditions.^10^

Notably, the absence of schistocytes, fragmented red blood cells typically present in TMA, on the peripheral smear was a key factor in differentiating this case from TMA. Furthermore, the patient's significant improvement following vitamin B12 supplementation further supported the final diagnosis of vitamin B12 deficiency‐induced hemolytic anemia.

While vitamin B12 deficiency is a well‐known cause of macrocytic anemia, its association with hemolysis is less commonly recognized.^4^

Our patient's clinical presentation and laboratory findings were consistent with those reported in similar cases. For instance, a case report by Tetali et al. described a 40 years caucasian female patient with severe anemia resulting from vitamin B12 and folate deficiency, who also exhibited non‐immune intramedullary hemolysis and was managed similarly with vitamin supplements resulting in improved clinical outcomes.^11^

The management of vitamin B12 deficiency involves replenishing the body's stores of this essential nutrient. In our patient's case, treatment with vitamin B12 led to a marked improvement in his condition. This aligns with the findings of Green, who emphasized the importance of timely diagnosis and treatment of vitamin B12 deficiency to prevent further complications, particularly neurological ones.^12^

Nonimmune hemolytic anemia must be included in the differential diagnosis when evaluating patients with anemia concurrent with vitamin B12 deficiency.^13^ Vitamin B12 supplementation stands out as the cornerstone in correcting this infrequent yet severe consequence of vitamin B12 deficiency, emphasizing the critical role of timely and targeted intervention in patient care. Vitamin B12 supplementation stands out as the cornerstone in correcting this infrequent yet severe consequence of vitamin B12 deficiency, emphasizing the critical role of timely and targeted intervention in patient care.

## AUTHOR CONTRIBUTIONS

**Ashish Tamang:** Conceptualization; data curation; formal analysis; investigation; methodology; project administration; resources; visualization; writing – original draft; writing – review and editing. **Prakash Sapkota:** Conceptualization; data curation; formal analysis; investigation; methodology; project administration; resources; supervision; validation; visualization; writing – original draft; writing – review and editing. **Ram Bahadur Gurung:** Conceptualization; data curation; formal analysis; investigation; methodology; project administration; resources; validation; visualization; writing – original draft. **Abiral Subedi:** Formal analysis; investigation; methodology; resources; writing – original draft. **Chhavi Sachdeva:** Writing – original draft; writing – review and editing.

## FUNDING INFORMATION

No funding was received.

## CONFLICT OF INTEREST STATEMENT

The authors declare that they have no conflict of interest.

## ETHICS STATEMENT

Written informed consent was obtained from the patient for the publication of the medical case details and any accompanying images.

## CONSENT

Written informed consent was obtained from the patient for the publication of this report following the journal's patient consent policy.

## Data Availability

The authors of this manuscript are prepared to provide supplementary information concerning the case report upon official request. All data generated or analyzed during this study are included in this article. Further inquiries can be directed to the corresponding authors.
